# The Effectiveness of Predicting Suicidal Ideation through Depressive Symptoms and Social Isolation Using Machine Learning Techniques

**DOI:** 10.3390/jpm12040516

**Published:** 2022-03-22

**Authors:** Sunhae Kim, Kounseok Lee

**Affiliations:** Department of Psychiatry, Hanyang University Medical Center, Seoul 04763, Korea; sunhk0906@hanyang.ac.kr

**Keywords:** social isolation, suicidal ideation, machine learning methods, depression

## Abstract

(1) Background: Social isolation is a major risk factor for suicidal ideation. In this study, we investigated whether the evaluation of both depression and social isolation in combination could effectively predict suicidal ideation; (2) Methods: A total of 7994 data collected from community residents were analyzed. Statistical analysis was performed using age, the Patient Health Questionnaire-9, and the Lubben Social Network Scale as predictors as the dependent variables for suicidal ideation; machine learning (ML) methods K-Nearest Neighbors, Random Forest, and Neural Network Classification were used; (3) Results: The prediction of suicidal ideation using depression and social isolation showed high area under the curve (0.643–0.836) and specificity (0.959–0.987) in all ML techniques. In the predictor model (model 2) that additionally evaluated social isolation, the validation accuracy consistently increased compared to the depression-only model (model 1); (4) Conclusions: It is confirmed that the machine learning technique using depression and social isolation can be an effective method when predicting suicidal ideation.

## 1. Introduction

Social relationships are decisive factors for lifelong development and emotional fulfillment [1]. Humans, as social beings, have a basic need to belong [2], and most of them live within a relational framework that defines their identity and personality [3].

Therefore, the effects of social relationships on individuals have been studied in various fields, ranging from physical and mental health to mortality and suicide [4]. In particular, since suicide is a major cause of death worldwide and occurs due to the complex interaction of biological, psychological, social, and situational factors, many studies on social relationships have been conducted. A variety of socio-relational factors characterize people who commit suicide; for example, suicide is more common among men and those who are single, separated, divorced, or widowed [5]. Data also show that suicide victims have fewer close friends [6] and are more likely to live alone than those who die of natural causes [7], indicating reduced opportunities for social interaction. Among those who attempt suicide, 22–25% report living alone [8,9], compared to 15% of the general population [8].

As such, it is important to understand individual social networks in suicide prevention [10]. Social networks measure the connections and interactions between individuals and their families, friends, colleagues, and neighbors, which have a lasting impact on an individual’s life [11,12]. Poor social support significantly increases the risk of suicide [13,14] and is also associated with lower levels of depression [15] and drug adherence [16,17,18]. In contrast, studies consistently show that low levels of social support from family predict suicidal behavior and ideation in adolescents [19,20,21]. Such low levels predict the likelihood of suicide by adulthood [22] and are also associated with poor adherence to treatment [23].

A sense of belonging may predict mental disorders, including depression, better than other components of social relations theory, such as social support [24]. A high sense of belonging is associated with promoting better social and psychological functioning [24], but a low sense of belonging is associated with loneliness [25], depression [24,26,27], and suicidal ideation [26]. As such, in those who attempt suicide, a frustrated sense of belonging causes them to experience negative emotions and psychological pain, which ultimately leads to suicide. A frustrated sense of belonging means feeling that you are no longer needed in your relationships with family members, friends, and other groups. This can be seen as a cause of suicide due to disconnection, isolation, and loneliness, which are expressed as a frustrated sense of belonging [28].

Social isolation is a broader concept than ’living alone’, and high suicide rates has been found [29]. Many researchers also found that when examining variables related to suicide rates, “the people who are most at risk of suicide are those who live isolated lives without contact with family, friends, or religious communities” [30,31,32,33,34,35]. Furthermore, there is the view that living alone has a strong influence on likelihood of suicidal success [36]. Most people who die by suicide feel a strong sense of isolation right before their suicide attempt [37]. Although people without suicidal tendencies can experience social isolation, a suicidal person experiencing social isolation is a strong predictor of suicide [38]. Consequently, social isolation is a major common factor related to suicide among adults [36,39,40,41,42,43] and the elderly [44,45,46,47] at all ages. Additionally, the concept of social isolation plays an important role in suicide theory [48].

The relationship between social isolation and suicidal ideation can be explained by three suicide theories. First, the traditional theory of suicide is ‘Interpersonal suicide theory’ by Joiner. According to the interpersonal theory of suicide, there are three components that an individual must experience to die by suicide: (1) the ability to engage in lethal self-harm (the desire to commit suicide), (2) perceived burdensomeness (belief that one is a burden on others or society), and (3) lacking a sense of belonging (feelings of isolation) [41]. Although an individual wishing to commit suicide is not necessarily capable of attempting suicide, the risk of a genuine suicide attempt increases if reduced fear of death and increased tolerance to physical pain are combined with the three components of suicide. Previous exposure to painful experiences will facilitate this process through habituation [41], and social isolation will further enable this process, leading to an increase in suicide risk.

Second, O’Connor’s (2011) ‘Integrated Motivational-Volitional model’ separates suicidal ideation and suicidal attempt. O’Connor suggests that defeat and entrapment are the primary motivators for suicidal ideation, and that impulsivity, access to lethal means, and abilities such as planning may explain why suicidal ideation acts in addition to them [49].

Finally, the ‘ideation-to-action’ framework of Klonsky and May (2014) expresses that development of suicidal ideation, and progress from ideation to suicide attempts viewed as distinct explanations. Suicidal ideation is caused by a combination of pain and hopelessness, where connecting with people is a key protective factor against heightening feelings of pain and hopelessness. In this theory, the progression from suicidal ideation to suicidal attempts occurs when these protective factors and other temperamental, acquired, and practical factors are high in the pain and fear inherent in the attempt to end life [50].

Therefore, according to the ideation-to-action theory, belongingness, an influencing factor in suicidal ideation, is important to prevent the progression to a more fatal suicide attempt. Klonsky (2014) also mentioned that belongingness to suicidal ideation can contribute to the pain and hopelessness that drive suicidal ideation [50]. The fact that the most frequently mentioned risk factors for suicide predict suicidal ideation but not suicidal behavior is very important, because most suicidal ideation individuals do not go through with suicide attempts [51]. Accordingly, a better understanding of suicide risk, particularly the progression from suicidal ideation to behavior, is important for both theoretical and clinical purposes in this field.

Suicide attempts and suicide completion have neurobiological correlations [52,53,54] and comorbidities with psychiatric disorders [55,56,57,58,59]. On the other side, understanding suicidal ideation requires a comprehensive assessment of an individual’s intrapersonal and interpersonal characteristics. While the main interpersonal characteristics of suicide risk are presented by social isolation, the key intraindividual characteristics include clinical diagnoses such as depression [60].

The following three psychological variables are considered particularly important predictors of suicidal ideations and attempts: depression, hopelessness, and impulsivity [61]. These variables represent statistically reliable relationships with suicidal ideation and suicide risk. Among them, depression is the strongest predictor of suicidal ideation [62].

Consequently, based on the theories about suicide so far, by taking the suicidal ideation, which predicts suicide risk at the forefront, as the dependent variable. This study focuses on social isolation, an important protective factor and depression as a strong predictor, for suicidal ideation. The purpose of this study is to investigate the influence of suicidal ideation prediction. Further, this study investigated whether the evaluation of social isolation and depression combined can effectively predict suicide using the results of a survey of local residents. To investigate this, differences between groups were compared and the accuracy of suicidal ideation was predicted using three machine learning techniques.

## 2. Materials and Methods

### 2.1. Participants

Of the total 8011 surveys in 2020 and 2021 in Yangpyeong, Gyeonggi-do, South Korea, this survey was conducted for the purposes of early detection and intervention in high-risk suicide groups in Yangpyeong-gun by Yangpyeong Mental Health Center, and a questionnaire survey was conducted for residents who agreed to participate in this project. Therefore, since the subjects who agreed to the survey completed the evaluation, most evaluation scales were performed. The implementation method was conducted by integrating Yangpyeong Mental Health center staff, public health center staff, and Gallup surveys (face-to-face and non-face-to-face surveys, e.g., face-to-face online survey and phone survey) only for those who agreed, owing to the COVID-19 pandemic.

In total, 7994 participants participated, excluding 16 where respondents did not provide basic information such as age and gender. Subjects agreed to participate in the study on condition of confidentiality, and a questionnaire was completed by consenting participants. The same content was implemented for two years, and the subjects surveyed in 2020 were excluded after identity verification so that the subjects of the investigation were not duplicated. It was a community cross-sectional survey, and no clinical information was obtained from the participants. This study was approved by the Institutional Review Board of Hanyang University Hospital (HYUHIRB-2022-01-043-001).

### 2.2. Measurements

#### 2.2.1. The Patient Health Quessionnaire-9 (PHQ-9)

The Patient Health Questionnarie-9 (PHQ-9) was a self-reporting questionnaire used as a depression screening tool [63]. The PHQ-9 was designed for the diagnosis of major depressive disorder in accordance with the nine items of the diagnostic criteria for major depressive episodes of the Diagnostic and Statistical Manual of Mental Disorders (DSM), and a total of nine items were used to evaluate the depressive symptoms. Each item ranged from 0 to 3 points, and the total score ranged from 0 to 27 points. A high score indicated severe depressive symptoms. In this study, the Korean version of PHQ-9 was used; its reliability and validity has been verified [64]. Cronbach’s alpha in this study was 0.933.

#### 2.2.2. Lubben Social Network Scale (LSNS)

The LSNS is a tool to evaluate social support and social isolation to measure the social network of the elderly [65]. In this study, (1) family networks, (2) friend networks, (3) confidant relationships, and (4) living arrangements were calculated on the small scale of LSNS. The subscale was used for comparison between groups, and in ML, the total score was used for model efficiency. A high score indicated good social relationships, and a low score indicated social isolation. The Korean version of LSNS was used; its reliability and validity have been verified. Cronbach’s alpha in this study was 0.720.

#### 2.2.3. Assessment for Suicide

In the Korean version of Mini International Neuropsychiatric Interview (MINI), a module for evaluating suicide risk was evaluated [66]. The MINI is a structured interviewing tool developed for the diagnosis of mental disorders. In previous studies, it was shown that the reliability and validity were high when compared to diagnostic systems such as DSM. A higher score indicated a higher risk of suicide. In this study, those who answered yes to questions 1–3 were selected as the suicide risk group, and the non-suicide risk group was used as the comparison group (Appendix A, Table A1). Cronbach’s alpha in this study was 0.695.

### 2.3. Statistical Analysis

The Student’s *t*-test and X^2^ test were used to examine the differences in the survey results between groups. To evaluate the prediction of suicidal ideation using machine learning, two prediction models were used: a model that evaluated only depression as a predictor (model 1) and a model that included social isolation (model 2). The age of each model was additionally corrected as a control variable. Accordingly, model 1 used age and the PHQ-9 total score as predictors, and model 2 used age, the PHQ-9 total score, and the LSNS total score as predictors.

As machine learning methods, K-Nearest Neighbors (KNN), Random Forest (RF), and Neural Network (NN) were used. For each model, 5116 data were classified as training data, 1280 as validation data, and 1598 as test data. All data were analyzed using JASP v0.16 (Amsterdam, The Netherlands) and MedCalc v20.022 (MedCalc Software, Mariakerke, Belgium).

#### 2.3.1. K-Nearest Neighbors Classification (KNN)

The KNN is a machine learning classification method that looks at the k predictor observations most similar to new observations to make predictions on class assignment. The number of nearest neighbors is intrinsically linked to the model complexity; the smaller the number, the greater the flexibility of the model [67].

#### 2.3.2. Random Forest Classification (RF)

RF is a classification method that generates a set of decision trees made up of a number of individual trees operating as an ensemble. Each individual tree in the RF returns a class prediction, and the class that receives the most votes becomes the prediction model [67,68]. In this analysis, variable importance was evaluated. Variable importance gives the degree to which each variable affects the accuracy of the model. When the predictive value of each variable is randomly excluded or replaced, depending on the degree of change in the performance of the model, if the replacement of each variable significantly changes the model performance, the importance of the variable increases. The partial dependence graph showed the contribution of each dependent variable to the independent variable in the form of a function of the variable, which indicated the change in the response variable according to the continuous change of each explanatory variable. At this time, the change of the response variable to the change of a specific variable was performed assuming that all other variables were average values.

#### 2.3.3. Neural Network (NN) Classification

Feedforward neural networks are prediction algorithms inspired by the biological neural networks that make up the brain. A neuron (node) that receives a signal can process the signal and send a signal to a connected neuron. The signal of a node is a real number, and the output of each node is calculated by sending a signal through an activation function. The number of layers and nodes in the network is intrinsically linked to model complexity, as it increases the flexibility of the model.

The NN technique sets an algorithm for network training. While the backpropagation option is standard for training neural networks, the other options are rprop+ (default) for elastic backpropagation with backtrace, rprop- for elastic backpropagation without backtrace, global-modifying the learning rate relative to the smallest absolute value. Gradient or gprop-slr was used as a global convergence algorithm that modifies the learning rate relative to the smallest learning rate itself [67]. Using the NN technique, it was possible to design a useful nonlinear system that accommodates a large number of inputs with a design based only on instances of input–output relationships [69].

## 3. Results

### 3.1. Experimental Results

#### General Characteristics

The mean age of the entire group was 56.41 years (SD = 16.57). In the group with suicidal ideation, the proportion of women was relatively high (33.3%). The mean age was significantly higher in the group with suicidal ideation, and the depression score was also higher (*p* < 0.001). The social relationship score was significantly higher in the group without suicidal ideation, and the four subscale scores were also high (Table 1).

### 3.2. Machine Learning Model Analysis

#### 3.2.1. Validation Accuracy of the Prediction Machine Learning Model

In the overall machine learning algorithm technique, the validation accuracy of the prediction model consistently increased when social isolation was additionally evaluated (model 2) compared to when only depressive symptoms were evaluated (model 1). In addition, the test accuracy was 0.9 or higher, indicating that it was excellent in all techniques (Table 2).

#### 3.2.2. Diagnostics Characteristics of Suicidal Ideation Using Machine Learning

**Figure 1:**
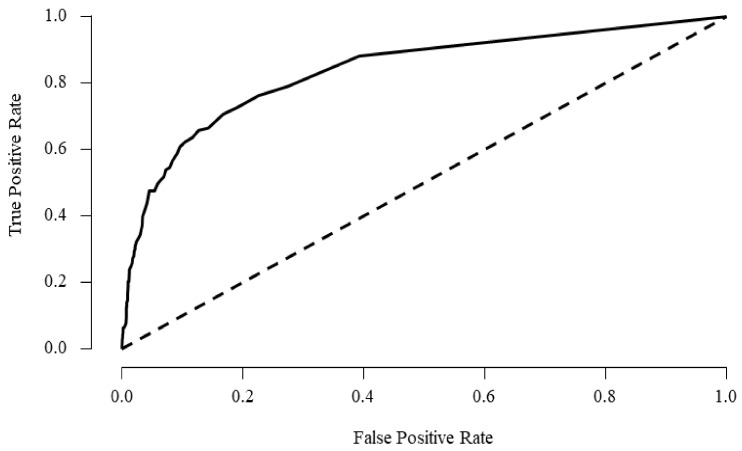
ROC curve plots for suicidal ideation using random forest (model 2).

Model 2 showed the highest AUC value (0.836) when the RF technique was used (Table 3, Figure 1). This is a significantly higher result than when social isolation is not used as a predictor (model 1). Similar results were shown when using the KNN technique (model 2 = 0.836 vs. model 1 = 0.645), but the NN technique showed the opposite result (model 2 = 0.643, model = 0.702, Table 3).

The suicidal ideation prediction of variables using depression and social isolation showed high specificity in all ML techniques (0.959–0.987), and RF model 2 predicted the highest suicidal ideation (AUC = 0.836, specificity = 0.987, 95% CI = 97.968–99.212, Table 3).

#### 3.2.3. Variable Importance in Random Forest Model

As a result of evaluating the importance of variables in the RF model 2, it was found that age was the next most important depressive symptom (0.023). The total increase in node purity was highest in depressive symptoms (0.236), followed by LSNS scores (Table 4).

## 4. Discussion

In this study, we investigated whether the evaluation of depression and social isolation combined could effectively predict suicidal ideation. The results showed significantly high AUC and specificity through the prediction of suicidal ideation using a ML technique, which was a useful technique for confirming groups with a high-risk of suicidal ideation.

The predictor model including social isolation (model 2) exhibited an improved validation accuracy, AUC, and specificity than model 1 in which only depression was set as a predictor variable. As a result, social isolation was confirmed as an important risk factor in predicting suicidal ideation, thereby indicating that depression and social isolation increase the risk of suicidal ideation and more accurately predict suicidal ideation. This integrates the most general concepts in previous studies that social isolation increases risk of suicidal ideation [13,14] and that social support has direct and indirect functions in the prevention and improvement of depression [26].

In this study, the RF algorithm, which is considered the most advantageous approach for predicting suicidal ideation among machine learning algorithms, was used. This is because it has the best test accuracy and F1 score in model 2 that measures suicidal ideation, which is advantageous for a predictive model compared with other machine learning algorithms (Table 2), and also has the highest AUC score in the diagnostic characteristic analysis of suicidal ideation (Table 3). In the RF model 2, which achieved the highest level of suicidal ideation prediction, depressive symptoms were the highest in variable importance and total increase in node purity, and social isolation and age varied according to each variable importance evaluation method. This may be due to the relatively high age of the entire group in this study, (~56 years). However, since this study was not conducted on the elderly specifically, it cannot be generalized to the elderly group. The study was conducted in Yangpyeong, characterized by a relatively large area, a small population, and a relatively large elderly population in Korea. These characteristics may be risk factors for suicide [70]. Although not as important as depressive symptoms, increasing age is an important variable in predicting suicidal ideation, which is consistent with the result of age having the second highest importance when evaluating variable importance in the RF analysis.

The limitations of this study are as follows, firstly, since this study is based on residents of one area, these results cannot be considered representative of the entire group. Secondly, gender was not considered. Due to the characteristics of NN, continuous variables are suitable for use as predictors. In order to apply a categorical variable such as gender to NN, methods such as conversion into a dummy variable or using a one-shot encoding method may be used. Accordingly, gender was added as a variable in the present analysis, however, results did not show significant improvements in accuracy. In future research, it is recommended to analyze various variables together using the accurate and excellent ability of machine learning techniques. Thirdly, the quality of interpersonal relationships could not be evaluated. The quality of interpersonal relationships can be determined by emotional sympathy as well as the structure of relationships and the degree of exchange, but they were not evaluated in the present study. Fourth, the PHQ-9 of the depression assessment includes a suicidal ideation item (item 9). Therefore, with respect to suicide, the dependent variable and the independent variable may overlap. Accordingly, an additional analysis was performed, except for item 9, but there was no significant difference (Appendix A, Table A2). In our previous study, there were some differences according to the ML technique [71], but the inclusion of item 9 did not indicate a difference in accuracy.

Nevertheless, this study tried to discriminate the depressive symptoms and social relationships most efficiently through ML techniques to predict suicidal ideation. According to the previous suicide theories, although social relationships are a major predictor of suicidal ideation, unfortunately, a social network review is often not included in the suicidal ideation assessment procedure, and therefore making it difficult to predict [72]. A machine learning technique is being recommended in psychiatry to improve the accuracy of diagnosis and prognosis, and to determine treatment methods [73]. In particular, in machine learning, the gold standard for model performance fits well with the model’s validation data [74,75,76,77], and it is also used in suicide prediction as it has an edge in accuracy and scalability compared with traditional statistical methods [78].

Thus, the concept of social networks can make a difference for those at risk of suicidal ideation. Social networks are particularly important when suicidal people are admitted to emergency rooms or discharged from psychiatric wards. Patients that are able to rely on supportive social networks can better adhere to prescribed treatment and cope with day-to-day difficulties. In particular, as this study was performed in the context of narrow social relationships in Korea in the COVID-19 pandemic, the possibility of higher social isolation exists. However, in a previous study of LSNS in Korea [79], the overall SD score was similar (male = 29.89, female = 27.62) to that in this study, and it was significant between the groups with and without suicidal ideation in this study. Therefore, social isolation is an important factor influencing suicidal ideation despite the involvement of environmental factors, such as COVID-19, when evaluating suicidal ideation risk, and assessment of social networks is recommended.

Social isolation is effective in determining suicidal behavior and discriminating between those who will commit suicide and those who will not [36]. Therefore, future research should investigate which of the types of suicide (e.g., suicidal ideation, suicidal attempt, suicidal behavior) are more characteristic of social isolation. In addition, since a decisive factor in preventing suicide is to relieve the pain of individual loneliness by social support [80,81] through meaningful relationships (e.g., parents, marriage, friends or romantic relationships) [82,83,84,85,86,87,88], and may contribute to suicide prevention and treatment.

This study confirms that social isolation and depression are effective for screening suicide. Most suicide screening tools measure suicidal ideation, attempts, and behavioral variables directly related to suicide. However, if the risk of suicidal ideation is evaluated using social isolation as a variable, it can be selected through an indirect description. In particular, it will have an advantage in identifying the risk of suicidal ideation in psychiatric outpatients, the elderly, and vulnerable groups with physical diseases, rather than the general population.

## 5. Conclusions

The RF technique was the most effective ML method in predicting suicidal ideation, and when considering social isolation in addition to depressive symptoms, the diagnosis accuracy was improved. These methods have high specificity and could be a useful tool to confirm suicide risk in a group with high suicide prevalence.

## Figures and Tables

**Table 1:** General characteristics and differences between groups.

Variables	Items	No Suicidal Ideation (*n* = 7214)	With Suicidal Ideation (*n* = 780)	t or x^2^	*p*
sex (male)		2820 (39.1%)	260 (33.3%)	9.854	0.002
age		56.08 ± 16.43	59.42 ± 17.55	5.071	<0.001
PHQ-9 total		1.86 ± 2.84	9.03 ± 6.59	30.082	<0.001
LSNS		30.26 ± 7.77	25.73 ± 8.29	14.573	<0.001
	family networks	8.91 ± 3.11	7.45 ± 3.26	11.949	<0.001
	friend networks	7.35 ± 3.45	5.62 ± 3.64	12.657	<0.001
	confidant relationships	5.37 ± 2.66	4.66 ± 2.86	6.655	<0.001
	living arrangements	2.13 ± 1.96	1.31 ± 1.75	4.764	<0.001

Values are presented as mean ± SD or number (%); PHQ-9: the Patient Health Questionnaire-9; LSNS: Lubben Social Network Scale.

**Table 2:** Accuracy in each predictive model.

ML Methods	Model	Validation Accuracy	Test Accuracy	Precision	Recall	F1 Score
KNN	model 1 ^1^	0.920	0.916	0.901	0.916	0.903
	model 2 ^2^	0.923	0.913	0.898	0.913	0.902
RF	model 1	0.905	0.907	0.896	0.907	0.900
	model 2	0.912	0.921	0.906	0.921	0.905
NN	model 1	0.909	0.921	0.912	0.921	0.911
	model 2	0.923	0.912	0.897	0.912	0.899

KNN: K-Nearest Neighbors Classification; RF: Random Forest Classification; NN: Neural Network Classification. ^1^ depression only predictor variable model; ^2^ depression and social isolation predictor variable model.

**Table 3:** Diagnostic characteristics of suicidal ideation assessments in each machine learning model.

ML Methods	Model	AUC	Sensitivity (%)	Specificity (%)	PPV (%)	NPV (%)
KNN	model 1	0.778	0.297 (22.504–37.787)	0.959 (94.812–96.892)	0.423 (34.061–51.006)	0.932 (92.475–93.816)
	model 2	0.830	0.325 (25.065–40.540)	0.974 (96.493–98.193)	0.570 (47.211–66.229)	0.933 (92.522–93.919)
RF	model 1	0.645	0.380 (30.382–46.027)	0.965 (95.448–97.412)	0.545 (46.129–62.710)	0.934 (92.620–94.128)
	model 2	0.836	0.252 (18.297–33.110)	0.987 (97.968–99.212)	0.655 (52.760–76.272)	0.931 (92.425–93.656)
NN	model 1	0.702	0.406 (33.136–48.375)	0.982 (97.426–98.864)	0.734 (64.250–80.911)	0.933 (92.462–94.023)
	model 2	0.643	0.313 (24.165–39.045)	0.979 (97.035–98.588)	0.625 (52.216–71.768)	0.928 (92.017–93.427)

AUC: Area Under the Receiver Operative Characteristic Curve; PPV: Positive, Predictive Value; NPV: Negative Predictive Value.

**Table 4:** Variable Importance in random forest analysis.

Variables	Mean Decrease in Accuracy	Total Increase in Node Purity
PHQ-9 total	0.0230	0.2360
LSNS total	−0.0005	0.0280
Age	0.0009	−0.0009

PHQ-9: The Patient Health Questionnaire-9; LSNS: Lubben Social Network Scale.

## Data Availability

The data presented in this study are available on request from the corresponding author. The data are not publicly available due to ethical restrictions.

## Appendix A

**Table A1:** MINI questions 1–3 items.

No.	Question
item 1	Think that you would be better off dead or wish you were dead?
item 2	Want to harm yourself or to hurt or to injure yourself?
item 3	Think about suicide?

**Table A2:** Accuracy in each predictive model (PHQ-9 used item 1 to item 8).

ML Methods	Model	Validation Accuracy	Test Accuracy	Precision	Recall	F1 Score
KNN	model 1	0.909	0.902	0.880	0.902	0.884
	model 2	0.900	0.907	0.888	0.907	0.892
RF	model 1	0.905	0.904	0.885	0.904	0.871
	model 2	0.905	0.911	0.895	0.911	0.896
NN	model 1	0.922	0.918	0.904	0.918	0.904
	model 2	0.916	0.911	0.896	0.911	0.896

KNN: K-Nearest Neighbors Classification; RF: Random Forest Classification; NN: Neural Network Classification. Model 1: depression only predictor variable model; Model 2: depression and social isolation predictor variable model.
